# Structural Characterization of Fibrils from Recombinant Human Islet Amyloid Polypeptide by Solid-State NMR: The Central FGAILS Segment Is Part of the β-Sheet Core

**DOI:** 10.1371/journal.pone.0161243

**Published:** 2016-09-08

**Authors:** Franziska Weirich, Lothar Gremer, Ewa A. Mirecka, Stephanie Schiefer, Wolfgang Hoyer, Henrike Heise

**Affiliations:** 1 Institute of Complex Systems, Structural Biochemistry (ICS-6), Research Centre Jülich, 52425, Jülich, Germany; 2 Institute of Physical Biology, Heinrich-Heine-Universität Düsseldorf, 40225, Düsseldorf, Germany; University of Pittsburgh School of Medicine, UNITED STATES

## Abstract

Amyloid deposits formed from islet amyloid polypeptide (IAPP) are a hallmark of type 2 diabetes mellitus and are known to be cytotoxic to pancreatic β-cells. The molecular structure of the fibrillar form of IAPP is subject of intense research, and to date, different models exist. We present results of solid-state NMR experiments on fibrils of recombinantly expressed and uniformly ^13^C, ^15^N-labeled human IAPP in the non-amidated, free acid form. Complete sequential resonance assignments and resulting constraints on secondary structure are shown. A single set of chemical shifts is found for most residues, which is indicative of a high degree of homogeneity. The core region comprises three to four β-sheets. We find that the central 23-FGAILS-28 segment, which is of critical importance for amyloid formation, is part of the core region and forms a β-strand in our sample preparation. The eight N-terminal amino acid residues of IAPP, forming a ring-like structure due to a disulfide bridge between residues C2 and C7, appear to be well defined but with an increased degree of flexibility. This study supports the elucidation of the structural basis of IAPP amyloid formation and highlights the extent of amyloid fibril polymorphism.

## Introduction

Islet amyloid polypeptide (IAPP or amylin) is a 37 amino-acid (aa) residue peptide cosecreted with insulin by pancreatic β-cells [1]. Physiological functions of soluble IAPP are related to regulation of gastric emptying and satiety control but not yet completely identified and understood [2]. IAPP is unfolded in its native monomeric state and has one of the most aggregation-prone amino acid (aa) sequences known [3]. IAPP constitutes the main component of amyloid deposits found in the pancreas of type 2 diabetes mellitus patients [4]. Although IAPP amyloid aggregates are not the cause of type 2 diabetes, they are associated with loss of mass and function of β-cells [5]. Furthermore, the failure of islet cell transplantation may in part be caused by rapid amyloid formation [4].

The aa sequence of IAPP is strongly correlated with its propensity to form amyloid fibrils. While human IAPP (hIAPP) is strongly amyloidogenic, mouse or rat IAPP does not form fibrils *in vivo* or *in vitro*. The aa sequences of the respective peptides differ in only six positions, five of which are located in the sequence region 20 to 29. Moreover, three of these aa residues in hIAPP (A25, S28 and S29) are substituted by a proline residue, a well-known β-sheet breaker, in non-amyloidogenic rat IAPP [6, 7]. This observation has initially drawn the attention to region 20 to 29 as being responsible for amyloid formation. Subsequent investigations on different short peptide fragments from the region of aa 20 to 33 of hIAPP showed that penta-peptides and longer segments are all able to form amyloid fibrils. However, the morphology of these fibrils and their aggregation kinetics strongly depend on the exact length of the fragment [8]. Furthermore, the short peptides hIAPP(22-27) and hIAPP(28-33) were crystallized and studied by high-resolution X-ray crystallography [9, 10]. While the segment hIAPP(28-33) forms an extended β-strand, for segment hIAPP(22-27) a backbone turn was observed in the crystal structure [10]. Amyloid fibrils from the segment hIAPP(20-29) were investigated also by solid-state NMR spectroscopy independently by two groups [11, 12]. Whereas in both cases extended β-strands spanning the full peptide length were found, the supramolecular arrangement in the two studies differs to some extent, most likely due to differences in sample preparation. Both samples contain fibrils with antiparallel β-sheets. However, in one study also a second fraction of fibrils with parallel β-sheets was detected [11], and homogeneous samples containing only fibrils with parallel β-sheets could be obtained by seeding the peptides with preformed fibrils derived from hIAPP(8–37), a construct lacking the N-terminal loop [11]. Apart from the central region 20 to 29, the segments hIAPP(8-20) and hIAPP(30-37) are also able to form fibrils [13, 14]. Interestingly, even the N-terminal loop can form non-amyloid fibers, as found by studies on the disulfide-linked fragment hIAPP(1-8) [15].

However, as the studies on fibrils of IAPP fragments and of other non-IAPP amyloidogenic proteins demonstrate, the structure as well as the supramolecular organization of amyloidogenic monomers in their amyloid fibrils critically depends on the full aa sequence [16]. Thus, structural elements determined from short model peptides may deviate substantially from those in fibrils from full length peptides. Furthermore, amyloid fibrils are prone to polymorphism, i.e. the morphology of the fibrils and the molecular structure of the monomers is not defined by the amino acid sequence alone, but may also critically depend on the exact fibrillation conditions, such as pH, salt concentration, fibrillation with or without stirring [17–21]. In addition, amyloid fibrils may be composed of more than one monomer with different conformations [22, 23].

A large number of structural studies on different fibril preparations from synthetic full-length hIAPP have been conducted in recent years. X-ray diffraction as well as electron diffraction, and cryo-electron microscopy confirm the cross-β structure with intermolecular β-sheets in which the β-strands are perpendicular to the fibril axis [24]. Electron paramagnetic resonance (EPR) spectroscopy revealed parallel, in-register alignment of the peptides, with the central region, comprising at least aa residues 12 to 29, being highly immobilized [25]. Solid-state NMR studies on full-length fibrils confirmed the parallel, in-register alignment of β-strands and suggested a hairpin structure of two β-sheets, which do not comprise the central region 18 to 27 [26]. Amide proton solvent exchange experiments suggested that regions 8 to 18 and 26 to 37 are solvent protected and thus involved in β-sheets [27]. In a second, more recent EPR study based on intramolecular distance measurements between two site-selective spin labels, no intersheet distances below 20 Å were detected [28], a finding which contradicts a close packing of β-strands as suggested by the steric zipper model for crystalline peptides [9]. Finally, the fibrillation of isotope labeled full-length IAPP was monitored by 2D Fourier transform infrared spectroscopy (FTIR) [29]. During the lag phase, a transient parallel β-strand appeared in the region aa 23 to 27, whereas N- and C-terminal β-strands were shown to form later in the process of fibrillation.

Based on all these studies, different structural models on full-length hIAPP have been built in recent years. They all agree on a parallel in-register alignment of β-strands. However, the number, position and relative arrangement of the β-strands strongly varies among these models. This may at least partly be due to the fact that different preparations may form different polymorphic structures, due to differences in fibrillation conditions. In addition to hairpin type structures composed of two β-sheets separated by a bend [10, 27, 28], alternative structural models, consisting of three β-sheets and two loops per molecule, have also been suggested [30, 31]. In particular, the conformation of the central segment hIAPP(23-27) differs substantially in the different models.

Here we present solid-state NMR data obtained from uniformly ^15^N, ^13^C isotope labeled fibrils from recombinant hIAPP with a non-amidated C-terminus, denoted as IAPP_COOH_ hereafter. Full site-specific resonance assignments were obtained, and an analysis of chemical shifts is performed. Resulting secondary structure elements in the sequence are shown as well as results pointing towards a well-defined but partly flexible N-terminus. Our data clearly indicate that the central segment hIAPP(23–28) is in β-sheet conformation in our fibril preparations of IAPP_COOH_.

## Experimental Procedures

### Recombinant expression and fibrillation of IAPP

Expression of human IAPP in *E*.*coli* was based on an engineered protein tag to prevent aggregation as described in detail by Mirecka et al. [32]. The β-wrapin was cleaved off by Protease Factor Xa digestion after expression and purification. With this expression protocol, the free acid form of IAPP, IAPP_COOH_ was obtained without any additional aa residues. Uniformly ^13^C, ^15^N-labeled IAPP_COOH_ was expressed and purified with a yield of about 3 mg pure peptide per liter of culture. It had an intact disulfide bridge between cysteine residues at positions C2 and C7. The oxidation state was proven by reversed-phase high-performance liquid chromatography (RP-HPLC) with different retention times for oxidized and reduced peptide forms [32]. Purified IAPP was lyophilized and stored at -80°C in glass tubes. Prior to fibrillation, IAPP_COOH_ was monomerized by dissolution in hexafluoroisopropanol (HFIP) to a concentration of 0.3 mM for 48 hours, lyophilized and stored at -80°C. Fibrillation was achieved in a stepwise procedure during seven consecutive days. At day one, a fraction of IAPP_COOH_ was dissolved to a concentration of 110 μM in fibrillation buffer at pH 7.4 (10 mM Na_2_HPO_4_/NaOH, 15 mM NaCl, 3 mM NaN_3_). The solution was exposed to intermittent sonication without shaking at room temperature overnight, as described previously in the literature [26]. The following six fractions were dissolved accordingly and added to the existing fibrillation solution in the sonication bath on a daily base. Turbidity of the solution was detected by unaided eye after few minutes. After fibrillation, the solution was centrifuged at 16,100 g and 4°C for 1 hour, followed by centrifugation at 100,000 g and at 4°C for 90 minutes. RP-HPLC analysis of the supernatant after fibrillation and pelletization revealed that monomeric IAPP_COOH_ was completely absent, i.e. the sample was completely fibrillated. After discarding the supernatant, the highly viscous sample was filled into a rotor from Agilent Technologies, with 3.2 mm diameter and 22 μl sample volume.

A second fibril sample was prepared accordingly by co-fibrillating ^13^C, ^15^N-labeled IAPP_COOH_ with unlabeled IAPP_COOH_ in the ratio 1:4.

### Atomic force microscopy

AFM on fibrillar IAPP_COOH_ was performed with a JPK NanoWizard II in Intermittent contact mode using an OMCL-AC160TS cantilever with a tip radius < 10nm. IAPP_COOH_ fibrils in fibrillation buffer were incubated at room temperature for one hour on a mica surface, washed with deionized water and dried with nitrogen gas. Images were taken with a line rate of 0.9 Hz and analyzed using JPK data processing software.

### Solid-state NMR experiments and processing of spectra

Magic-angle spinning (MAS) NMR experiments were performed at Varian spectrometers at fields of 14.1 Tesla and 18.8 Tesla (Experimental details of all experiments are given in S1 Table). All but one pulse sequences used started with an initial ^1^H to ^13^C cross-polarization step [33]. Exception was the J-coupling based INEPT (*I*nsensitive *n*uclei *e*nhanced by *p*olarization *t*ransfer) experiment [34]. An external calibration of the sample temperature, using the ^1^H chemical shift of nickelocene as a temperature standard [35], showed that at a spinning frequency of 11 kHz the actual sample temperature was 10°C ± 3°C above the temperature of the variable temperature (VT) gas which in the following is given as the nominal sample temperature.

Proton-driven spin-diffusion (PDSD) experiments were conducted with longitudinal mixing times ranging from 20 ms to 200 ms [36]. Mixing times of up to 50 ms mainly led to intra-residual cross-peaks in spectra, while inter-residual peaks showed up with longer mixing times. A 200 ms mixing time PDSD was performed with MAS spinning of 12,500 Hz at an 800 MHz proton frequency spectrometer. This spinning frequency was set close to a Cα-CO rotational resonance condition and enabled detection of weak inter-residue couplings *C*′(*i* − 1) → *Cα*(*i*) in vicinity of strong intra-residue couplings [37]. The observed inter-residual cross-peaks were strong indicators for sequential linking of residues and were used for sequential resonance assignment. For discrimination of one-bond correlations from relayed and sequential ones, SPC5_3, a double quantum coherence sequence, was used at an MAS spinning frequency of 11 kHz [38].

A further ^13^C, ^13^C correlation experiment used was based on a homonuclear double-quantum transfer step called DREAM (*D*ipolar *r*ecoupling *e*nhanced by *a*mplitude *m*odulation) [39]. DREAM mixing was done with a tangentially shaped soft pulse during 1500 μs, followed by a 90° pulse on ^13^C nuclei.

In the 2D and 3D ^15^N, ^13^C correlation experiments, the 2^nd^ transfer relied on spectrally induced filtering in combination with CP (SPECIFIC-CP) and enabled a frequency selective polarization transfer from amide to Cα or CO nuclei [40]. NCACX and NCACB spectra served the assignment of ^15^N backbone shifts and NCOCX the linking of residues, as magnetization was transferred from *N*(*i*) → *C*′(*i* − 1) and further to *CX*(*i* − 1) nuclei by spin diffusion.

The NHHC experiment [41] was recorded at a spinning rate of 11 kHz, with CP contact times of 200 μs, 200 μs and 70 μs μs for HN, NH and HC transfers respectively, and a longitudinal proton mixing time of 50 μs.

High-power broadband decoupling on protons with SPINAL phase modulation [42] was applied for all spectra during acquisition and *t*_1_, *t*_2_ evolution times. All processing of raw data was performed with NMRPipe [43]. Spectra were multiplied with sine-bell apodization functions shifted from 0.25 π in ^13^C dimensions to 0.4 π in ^15^N dimensions. Spectra were analyzed using CcpNmr Analysis [44].

The low temperature spectrum of fibrillated IAPP_COOH_ was acquired with dynamic nuclear polarization (DNP) enhancement [45] on a 600 MHz Bruker Avance III HD spectrometer at a sample temperature of ~100 Kelvin. The gyrotron provides microwaves at 395 GHz frequency. For better freezing properties and optimization of the DNP enhancement, the final sample contained 10% H_2_0, 30% D_2_O and 60% d_8_-Glycerol. The biradical added to the sample was AMUPOL [46] to a final concentration of 20 mM. The double-quantum DNP spectrum was recorded at 8 kHz MAS and with SPC5_2 recoupling [47].

### TALOS-N secondary structure prediction

Experimentally derived ^13^C and ^15^N chemical shifts were exported from CcpNmr Analysis for a TALOS-N prediction [48]. Based on secondary chemical shifts and sequence information, TALOS-N empirically predicts protein backbone torsion angles and a measure of rigidity expressed as RCI S^2^ value. No dihedral angles are predicted for residues at first and last position in the sequence. Resulting from the analysis were 35 pairs of dihedral angles φ and ψ, of which 30 pairs were classified as strong predictions and 5 as ambiguous. Ambiguous predictions resulted for residues N3, T4, N21, S29, and G33. These were all not part of a β-strand. The dihedral angles are found in S3 Table. Residues were designated as part of a β-strand if TALOS-N predicted the secondary structure with a probability higher than 75%.

## Results

### Recombinant expression and fibrillation

Uniformly ^13^C, ^15^N-labeled human IAPP_COOH_ was expressed recombinantly in *E*.*coli* as a fusion construct with an engineered β-wrapin to prevent aggregation during expression and purification [32]. The β-wrapin was cleaved off proteolytically by factor Xa treatment and removed together with the added protease by RP-HPLC. With this protocol, full-length hIAPP was obtained without any additional amino acid residues. However, in contrast to naturally occurring hIAPP which is C-terminally amidated (IAPP_CONH2_), the free acid form (i.e., without the C-terminal amide group) of hIAPP (IAPP_COOH_) was obtained.

**Fig 1:**
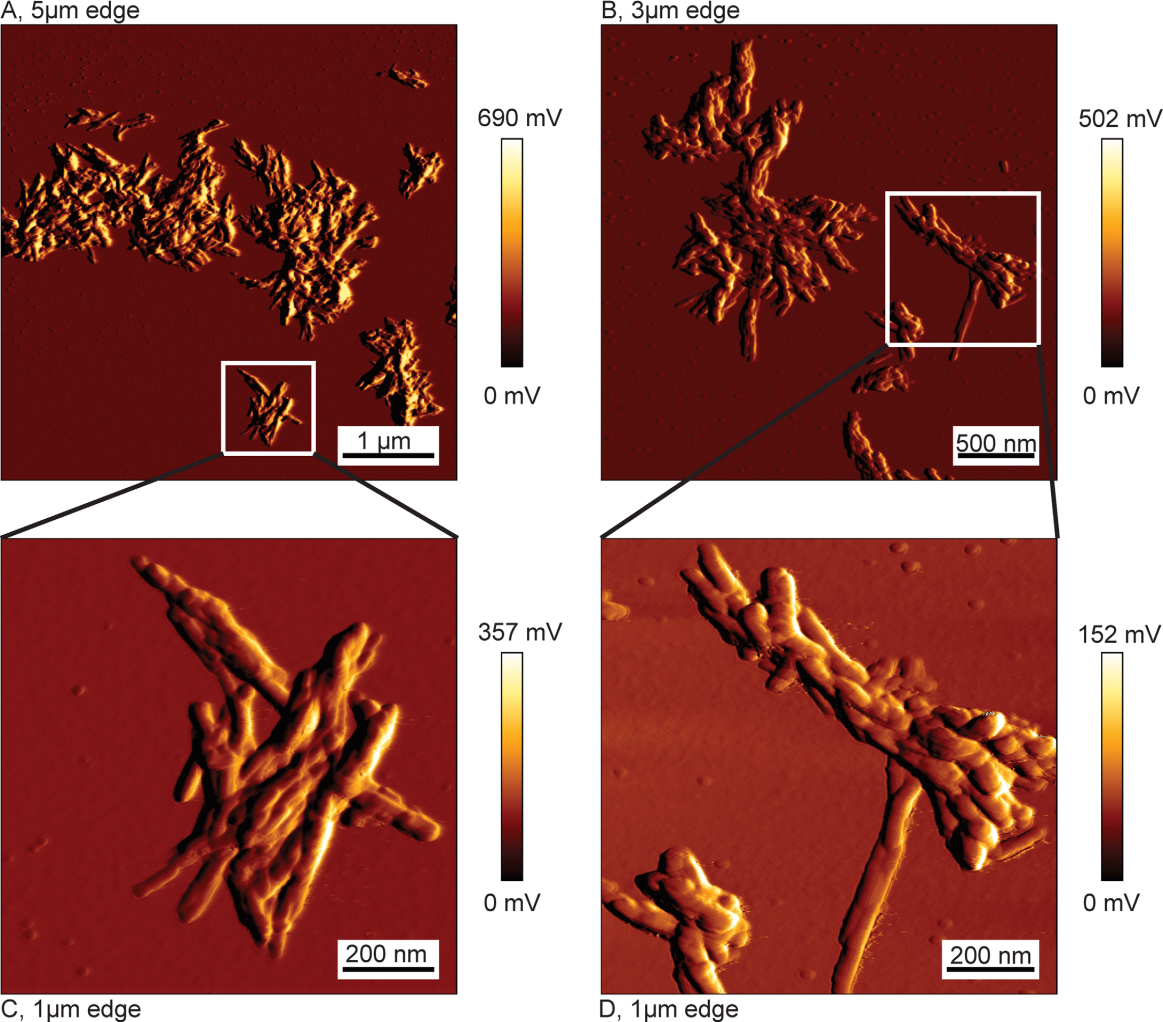
Atomic force micrographs of IAPP after fibrillation. Shown are scans of two different areas of the sample (A, B). C and D are additional scans focusing on smaller regions of A and B. Each scan consists of 1024x1024 pixels. Fibrils appear to laterally assemble into bundles.

Prior to fibrillation, IAPP_COOH_ was monomerized by dissolution in HFIP. Seeded fibrillation of pure, monomeric IAPP_COOH_ was conducted with intermittent sonication at pH 7.4 as described before [26]. Spontaneously formed aggregates served as initial seeds. Atomic force microscopy (AFM) of the fibrils revealed that the monomeric IAPP_COOH_ sample was converted to short fibrils which were laterally assembled into bundles, which tend to be attached to each other (Fig 1). This fibril morphology was typical for our sample preparation and could be reproduced in multiple fibrillation assays [32], although it decidedly differs from fibril preparations of other groups [26, 49]. Other aggregate types, like amorphous aggregates, were not detectable. As no single fibrils were observed, variations in thickness of the fibril bundles did not allow any conclusions about the sample homogeneity, and sample polymorphism can neither be confirmed nor ruled out from the AFM images alone.

### Site-specific resonance assignment

Solid-state MAS NMR experiments were performed at magnetic fields of 14.1 Tesla and 18.8 Tesla on a uniformly ^13^C, ^15^N-labeled sample. In cross-polarization based spectra [50], exhibiting only the rigid parts of the sample, line-widths ranging from 0.8 ppm to 1.3 ppm in the ^13^C dimension and below 3 ppm in the ^15^N dimension were observed for well-resolved signals in 2D spectra. These line-widths are comparable to those observed for spectra of other fibril preparations of non-functional amyloids [18, 26, 49, 51, 52], suggesting that the rather inhomogeneous appearance of the laterally assembled bundles in AFM images does not substantially affect the structure of the monomers on the molecular level. In an initial INEPT experiment [34], performed at a sample temperature of ~ 10°C, no signal was observed. The lack of signal in this J-coupling based transfer scheme indicates that all 37 amino-acid residues of the peptide, including the N-terminal disulfide-bridged loop, lack high flexibility [18, 53].

**Fig 2:**
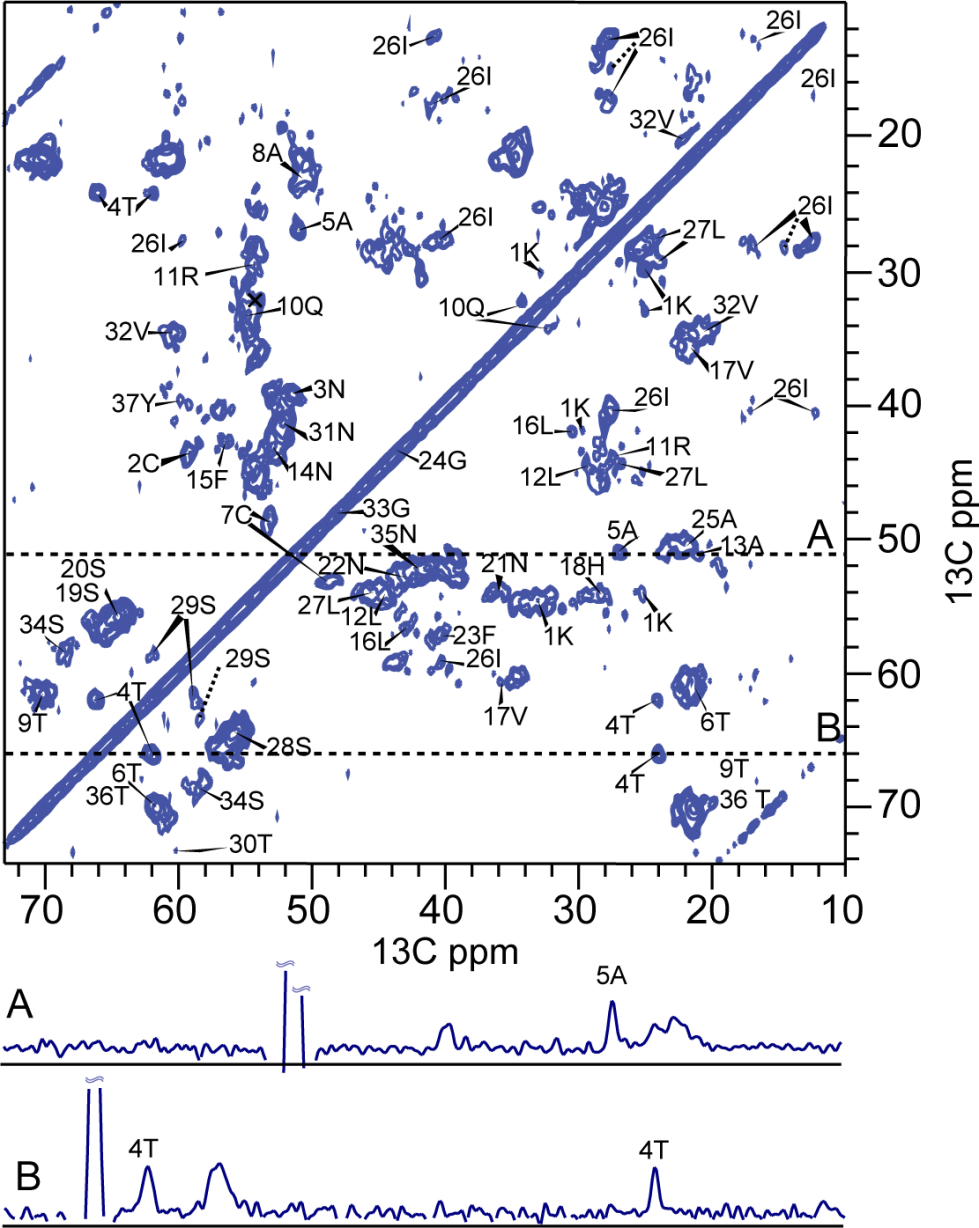
Proton-Driven-Spin-Diffusion (PDSD) spectrum. The spectrum was recorded at a field of 18.8 Tesla with longitudinal mixing time of 20 ms and spinning speed of 11 kHz. Sequential assignments shown are based on a number of experiments and brought together in this figure. There are two cross-sections drawn underneath, taken at the positions indicated by dotted lines. The dashed pointers mark the additional peaks found for I26 (Cδ_1_) and S29 (Cβ).

**Fig 3:**
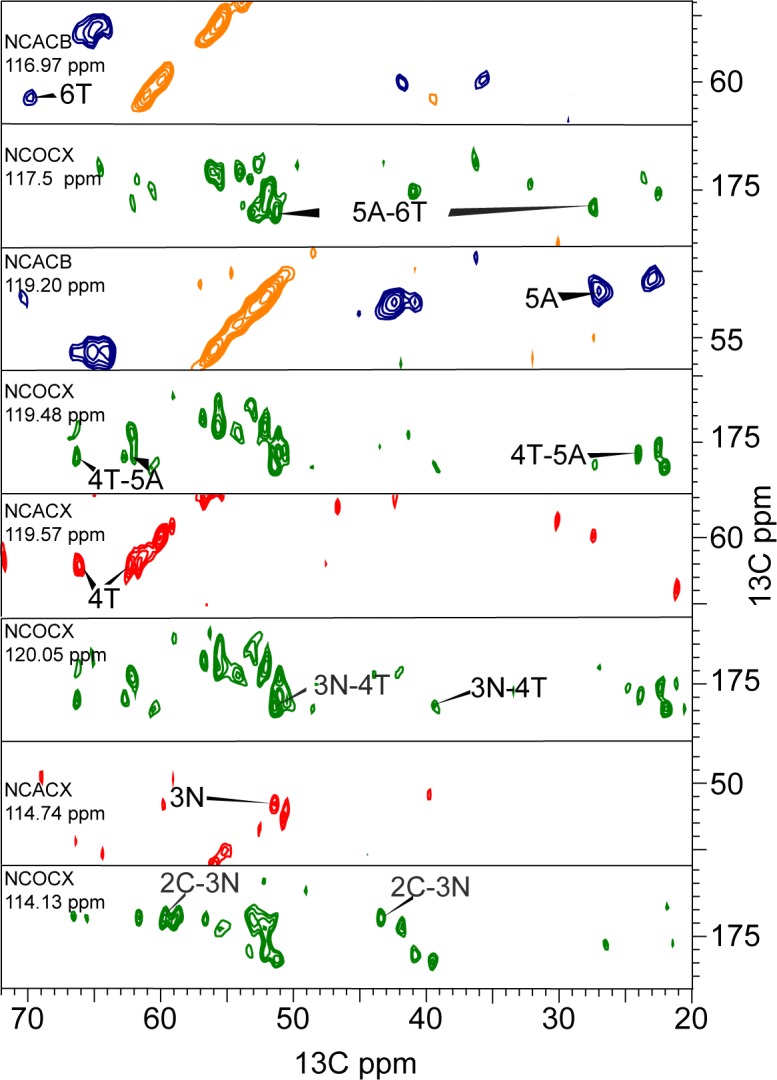
Sequential walk in the N-terminus via backbone nitrogen chemical shifts. The orange and blue peaks belong to a 3D NCACB spectrum (positive, negative) acquired at 14 kHz MAS at 14.1 Tesla. Red peaks belong to a NCACX 3D spectrum recorded at 14.1 Tesla and 11 kHz MAS spinning. Green peaks come from a 3D NCOCX experiment acquired at the same spectrometer at 14 kHz MAS spinning. All experiments were performed at a nominal sample temperature of 10°C. Reading from up to down, a spin system *i* is assigned to its backbone nitrogen shift in the upper strip. In the strip underneath, the preceding residue (green peaks) *i-1* appears at the same nitrogen shift as found above. Deviations of up to 0.7 ppm appear due to the line-widths of the nitrogen chemical shifts.

Full sequential resonance assignments were obtained from a set of 2D and 3D ^13^C, ^13^C and ^15^N, ^13^C correlation experiments (S1 Table and S2 Table) [37, 39, 40]. In Fig 2, the aliphatic region of a PDSD spectrum with 20 ms longitudinal mixing time is shown, and resonance assignments are indicated. Most of the 37 aa residues gave rise to one single set of resonances, and site-specific resonance assignments could be obtained from inter-residual cross correlations. These were observed in PDSD spectra recorded close to the rotational resonance condition between the CO and the Cα region (S1 Fig) [37]. Furthermore, inter-residual cross correlations were obtained from a combination of 3D NCOCX, NCACX, and NCACB spectra. The ^15^N, ^13^C correlation spectra were used for a sequential walk along the backbone amide for sequential assignment (Fig 3).

The majority of residues gave rise to intense cross-peaks in PDSD spectra, which points to strongly dipolar coupled spins and thus a rigid conformation. Less intense Cα-Cβ cross-peaks appeared for amino-acid residues L16, V17, I26, T30, S34, and Y37. At these sites, the conformation might be less constrained than at sites which showed more intense peaks, thus either resulting in higher flexibility or increased conformational heterogeneity. At position I26, the Cα-Cβ cross-peak was scattered over a range of 1.5 ppm. Likewise, for S29, a slight degree of peak doubling, resulting from some scatter of the Cβ chemical shift was observed. The main peak was observed at Cβ of 61.7 ppm, but a minor conformation also showed a Cβ shift of 63.7 ppm. This suggests some residual disorder in the region 26-29. Furthermore, for I26, two distinct C(_1_ signals, an intense (12.4 ppm) and a weak one (14.4 ppm), were observed. The weak signal is marked by a dashed line (Fig 2). While a shift of ~14.8 ppm or higher is found to be typical of a 100% population of the all-trans conformation of the χ_2_ dihedral angle [54], a shift of 12.4 ppm is close to the average chemical shift value determined by solution NMR [55], which is indicative of a free rotation around the bond connecting Cβ and Cγ_1_. Thus, for a small fraction of monomers, the I26 sidechain appears to be rotationally constrained to one conformation, while free sampling of the χ_2_ space seems possible for the larger fraction.

Residue S34 is the only residue that was completely absent from the 3D NCOCX spectrum which was recorded using a weak spin-lock on ^13^C (~5 kHz). This also points towards a different degree of flexibility at this site as apparent from accelerated T_1ρ_ relaxation (see below). Its neighboring residues G33 and A35 were however well observed in this experiment.

### Residues of the N-terminal loop are well ordered, but dynamic

**Fig 4:**
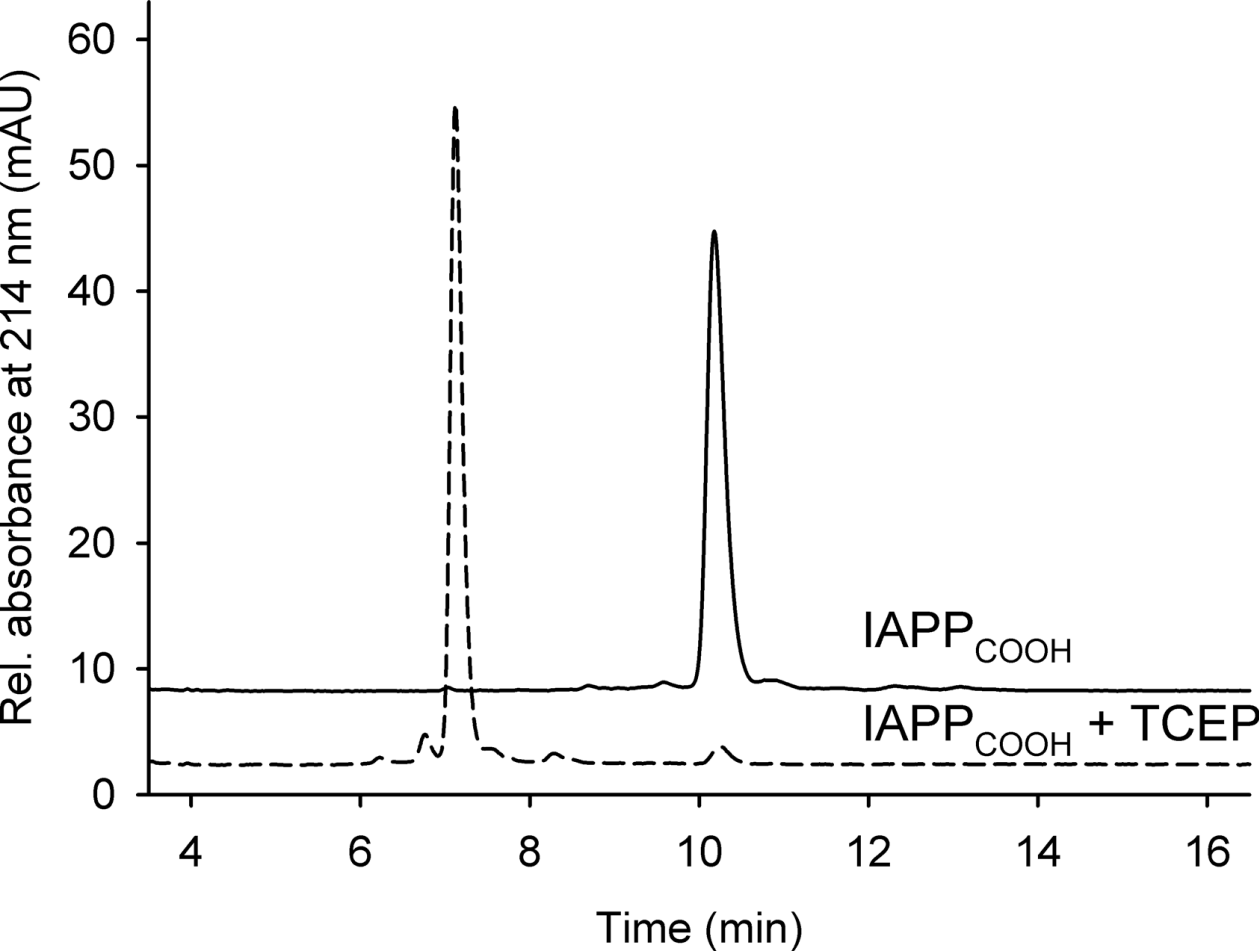
Analytical RP-HPLC before fibrillation. Performed under reducing (dashed line) and oxidizing (solid line) conditions to verify the presence of the disulfide bridge between residues C2 and C7.

Surprisingly narrow ^13^C line-widths of 0.8 ppm and intense peaks were observed for the N-terminal aa residues N3 to T6, which, together with C2 and C7, form a disulfide bridged N-terminal loop. The presence of the intra-molecular disulfide bridge was confirmed by RP-HPLC, based on different retention times for oxidized and reduced IAPP, before fibrillation (Fig 4). As a result of the loop structure, the chemical shifts of N3, T4, and A5 differed decidedly from those of the same residue types in the remainder of the sequence. In particular, the Cβ resonance of A5 exhibited a strong positive shift to a value of 26.8 ppm. The oxidation state of the disulfide bridge also strongly influenced the Cβ chemical shifts of the two cysteine residues [56]. Their Cβ shifts were highly shifted to values of 43 ppm and 49 ppm.

**Fig 5:**
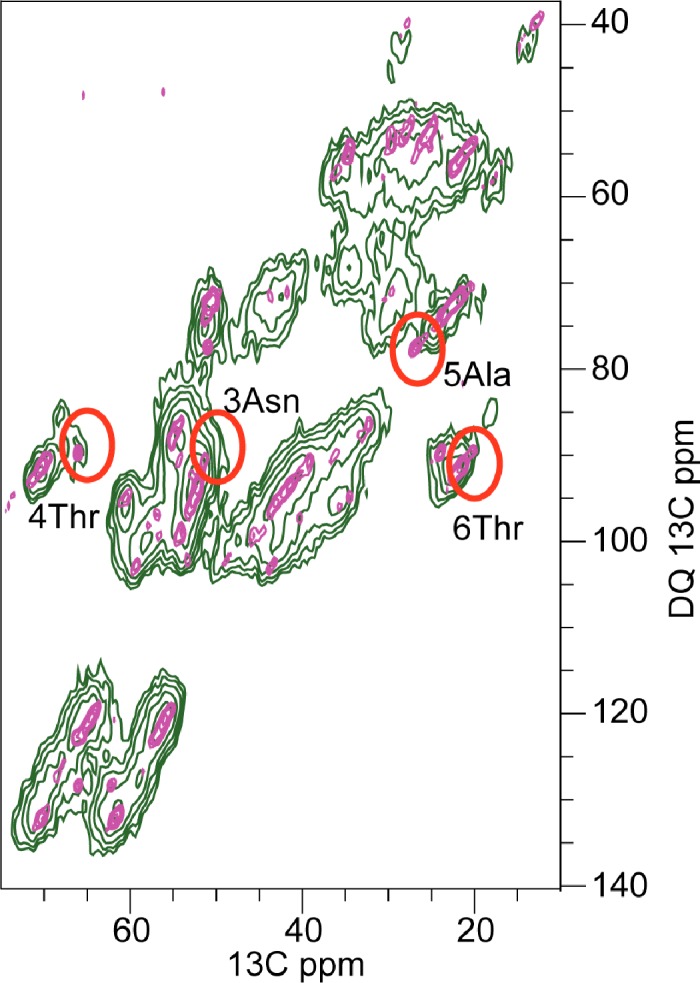
The effect of low temperature and hyperpolarization on the sample. Overlay of 2D Single-quantum double-quantum spectra recorded at magnetic fields of 14.1 T. The green spectrum was acquired with Dynamic-Nuclear Polarization (DNP) at 100 Kelvin nominal sample temperature and SPC5_2 recoupling at 8 kHz MAS. The purple spectrum was acquired with conventional solid-state NMR technique at 0°C nominal sample temperature with SPC5_3 recoupling at 11 kHz MAS. In the DNP experiment, the sample was frozen out and it is obvious, that the N-terminal Cα-Cβ cross-peak are broadened due to impeded molecular motion.

The signal intensities for residues N3, T4 and A5 varied in ^15^N, ^13^C correlation experiments, depending on the ^13^C spin lock field applied during the second cross polarization step. For radiofrequency-fields on ^13^C with a nutation frequency below 7 kHz, ^15^N, ^13^C cross correlation signals appeared weak, whereas for spin-lock fields with a frequency higher than 15 kHz, the intensity was as good as for other residues. This enhanced T_1ρ_ relaxation at low spin-lock fields may be an indication of additional slow dynamics of the N-terminal loop in contrast to the other residues in the sequence [57]. At extremely low temperatures around 100 K, when all residual motions are frozen out, signals of NMR spectra are inhomogeneously broadened by the resulting conformational disorder. In Fig 5, an overlay two ^13^C, ^13^C double quantum correlation spectra of fibrillar IAPP at ~10°C and at 100 K is shown. While the signals of all residues are affected by line broadening, this effect is most pronounced for residues of this loop. In particular, the line-broadening for the cross correlations of the Cα-Cβ correlation of aa residue A5 is broadened beyond detectability. A similar effect has been observed in a previous study by Luca et al., where lyophilization of the sample had the same effect [26].

### Sample homogeneity and reproducibility

A second fibril sample was prepared under identical conditions for expression, purification and fibrillation. Again spontaneous aggregates served as initial seeds. In contrast to the first sample, one part of uniformly ^13^C, ^15^N-labeled IAPP_COOH_ was mixed with four parts of unlabeled IAPP_COOH_ prior to fibrillation. The first and the second sample were analyzed in 2D ^13^C, ^13^C correlation spectra to compare their chemical shifts (S2 Fig). The cross peaks of most residues coincided, which indicates that two independent fibrillations under equal conditions resulted in fibrils of the same molecular structures, as a different conformation of monomers would result in shifted peaks. Very prominent were again the narrow peaks from the N-terminal residues N3 to T6. They coincided in both samples and again showed intense signals. Thus, also the N-terminus appears to be in the same conformation or cover the same conformational ensemble in both samples.

Like in the first sample - the non-diluted uniformly ^13^C, ^15^N-labeled IAPP_COOH_- peak doubling for S29 (Cβ) and I26 (Cδ_1_) was also observed in the second sample. However, the ratios of the signals differed between the two samples, thus ruling out the existence of complex fibrils consisting of monomers with different conformations as observed in [22, 23]. In contrast to the first sample, for S29, the signal with the Cβ shift of 63.7 ppm dominated in the spectrum of the second sample. Likewise, for I26, a Cδ_1_ chemical shift of 14.8 ppm, indicative of the rigid trans-conformation, was more pronounced than the shift of 12.4 ppm resulting from rotational averaging.

Peaks from two additional alanine and threonine spin systems, which could not be integrated into the sequence by means of a sequential walk, were observed mainly in a DREAM spectrum (S3 Fig) [39]. We interpret these peaks as a certain amount of impurity in the sample which was not eliminated through the process of purification.

### Secondary structure analysis

NMR chemical shifts strongly correlate with molecular conformation [58, 59]. We calculated the differences of secondary chemical shifts as Δ*δ C*_sec_ = Δ*δ C*_α_- Δ*δ C*_β_, with Δ*δ C*_x_ = *δ C*_x_(exp) - *δ C*_x_(BMRB) [60]. For the aa residues C2 and C7, Cβ random coil values of 40.7 ppm were used in the calculation to take into account the oxidized disulfide bridge [61].

**Fig 6:**
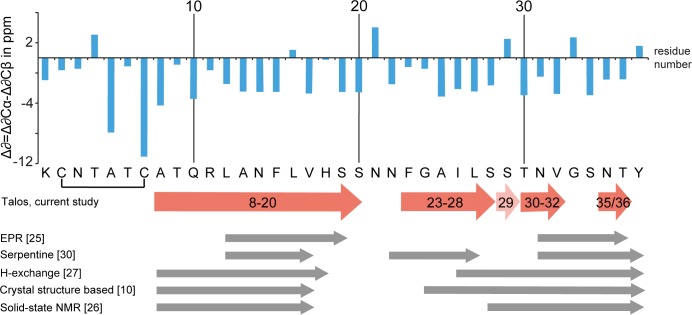
The location of β-strands in fibrillar IAPP. Top, calculated differences of secondary chemical shifts Δ*δ C*_sec_ = Δ*δ C*_α_- Δ*δ C*_β_, with Δ*δ C*_x_ = *δ C*_x_(exp) - *δ C*_x_(BMRB). Two or more adjacent negative values usually are indicative of a β-strand. Bottom, red arrows represent the β-strands predicted by TALOS-N based on NMR chemical shifts from the current study (except for residues A5, T6 and C7, which are part of the disulfide bridged N-terminal loop). Position S29 is found to be structurally less constrained and able to form part of a β-strand in a fraction of fibrils. Grey bars indicate the location of β-strands as determined in previous studies.

Three or more adjacent negative values indicate an extended conformation typical of β-sheets. In Fig 6, the secondary chemical shift values are displayed as blue bars. As expected for an amyloid fibril, the whole peptide shows a high degree of β-sheet secondary structure, with only six residues showing a positive value.

For a more detailed analysis of secondary structure, all experimentally derived ^13^C and ^15^N chemical shifts were used for a TALOS-N prediction of backbone torsion angles (S3 Table) [48]. Sites for which TALOS-N predicted torsion angles typical for β-strands with a prediction classification as “strong” were interpreted as being part of a β-strand. An exception was made for aa residues A5, T6 and C7, for which extended conformation was predicted by TALOS, but which cannot be part of an extended β-strand due to their location in the disulfide-bridged loop and were thus omitted from the first β-strand. According to this analysis, the fibrillar peptide comprises three to four β-strands, i.e. depending on the Cβ shift used for S29, the second and third β-strands are merged to one single strand or not. The strands are located at aa positions 8 to 20, 23 to 28, 30 to 32, and 35 to 36, as displayed in Fig 6. They are interrupted by one or two residues with random coil like conformation. Fig 6 also includes a comparison of the findings of our TALOS-N secondary structure analysis with results from former studies [10, 26–28, 30]. Secondary chemical shifts and TALOS-N results are in agreement, meaning that at positions with positive values, also the β-strands predicted by TALOS-N are interrupted. The only exception is L16, for which TALOS-N predicts torsion angles φ = -76°, ψ = 132° with standard deviations of 11° and 8°, although this residue has a small positive secondary chemical shift of 0.7 ppm. For this aa residue, as well as for the neighboring aa residue V17, Cα-Cβ cross-peaks were less intense than for most other residues assigned to β-strands, either because of increased flexibility or increased conformational disorder, an observation which may point to a short perturbation of the β-strand around positions 16/17. However, TALOS-N predicts for both residues torsion angles which are characteristic of β-sheet conformation.

TALOS-N predictions of backbone angles were classified as “strong” for all β-strand regions including also residues 16/17 and ambiguous at the kink positions N21, S29, and G33, as well as for residues N3, T4 in the N-terminus. The kink residues, connecting the C-terminal two or three β-strands, exhibited specific features. At position S29, depending on the Cβ chemical shift used, the prediction was either random coil (61.7 ppm) or β-strand (63.7 ppm). The second and third β-strand might thus be merged to one single β-strand spanning residues F23 to V32. A further reason for the shifted Cβ chemical shift could be that the side-chain hydroxyl group forms a hydrogen bond, either to the protein backbone [62], or to an adjacent serine residue in an intermolecular polar zipper motif [10].

For residue G33, the predicted dihedral angles were widely scattered in the Ramachandran plot. No structural prediction was made based on these angles. As described above, S34 appeared more dynamic due to its divergent behavior compared to its neighbor N35 in NCOCX with weak spin-lock on ^13^C. We thus interpret G33 and S34 as having a more dynamic behavior than the adjacent β-strands and do not ascribe them as part of such.

To further corroborate the extended conformation of the core region predicted by secondary shifts, we performed NHHC experiments[41] with longitudinal mixing times of 50 μs. In extended β-structures, Hα(i) nuclei and HN(i+1) are close in space [63] and should thus yield the strongest cross peaks in a 2D NHHC spectrum for short longitudinal proton mixing times < 100µs [64]. In S4 Fig, sequential N(i+1)-Cα(i) cross-peaks from assigned chemical shifts are plotted for all aa residues of the predicted core regions 8–20, 23–28 and 30–36 and overlaid with an NHHC spectrum obtained with a longitudinal mixing time of 50 μs. Signal overlap due to limited resolution in the indirect dimension prevents an unambiguous resonance assignment of resonances. However, all interresidual N(i+1)-Cα(i) cross-peaks predicted for residues in the β-strands agree well with the spectrum, as well as most of the N(i+1)-Cβ(i) cross-peaks.

## Discussion

### Polymorphism in IAPP fibrils

Polymorphism is a feature frequently observed for disease-related amyloid fibrils[17–21]. Polymorphism often becomes evident when different fibril preparations are compared, but can also occur within a single fibril preparation. AFM images of all samples of fibrillated IAPP_COOH_ revealed short fibrils which were laterally assembled into bundles, consisting of multiple filaments. Thus, no conclusions about sample homogeneity could be drawn from AFM images alone. Solid-state NMR experiments on fully ^13^C, ^15^N-labeled fibrillar IAPP_COOH_, however, yielded spectra showing a single set of resonances for the majority of aa residues, indicative of high sample homogeneity with a common, rather well-defined conformation of IAPP molecules incorporated into the fibril.

Inter-sample polymorphism can be caused by differences in the chemical identity of the amyloidogenic protein (e.g., aa sequence or posttranslational modifications) or differences in the fibrillation conditions. To evaluate polymorphism between different IAPP fibril preparations, we compared our data with solid-state NMR data reported by Luca et al. on solid-phase synthesized IAPP_CONH2_ with a residue-specific ^13^C labeling scheme for unambiguous resonance assignments [26]. An overlay of the chemical shifts obtained from this study and our PDSD spectrum is shown in S5 Fig Major differences in chemical shifts were observed for the region spanning residues L27 to S34. For residue A5 and the region A8-I26, chemical shifts were in reasonable agreement. The unusual chemical shifts of A5 were identical in both studies. Moreover, residue N21, whose Cβ chemical shift is 4 ppm less than those of the other asparagine residues, appeared in both studies at the same position in the spectra.

The overlay shows that chemical shift differences between both studies did not arise from disagreements in sequential assignments. For residues L27 to S34, cross-peaks appeared at different positions in the two spectra. This difference was very pronounced for serine residues, where three out of a total of five cross-peaks appeared at different positions (S28, S29 and S34). From the partial mismatch of spectra, we conclude that we have studied a different polymorph of IAPP fibrils that showed significant chemical shift differences in the aa region L27 to S34. These differences in chemical shifts might be a consequence of the difference in the C-terminus, which was amidated in the study of Luca et al. and occurred as a free acid in our study. They could, however, also reflect IAPP polymorphism independent of C-terminal (de)amidation and stem from alterations of the fibrillation pathway caused by subtle differences in the fibrillation conditions.

### FGAILS-region forms a β-strand in IAPP_COOH_

Structural characterization of the monomers in fibrils by chemical shift analysis led to the result that aa regions 8 to 20, 23 to 28, 30 to 32, and 35 to 36 form β-strands. Our data agree with previous models on the presence of a single β-strand in the N-terminal half of the peptide sequence (Fig 6), however with variations regarding its exact length and position [10, 26–28, 30].

More profound differences appear in the C-terminal half of the peptide. This half contains the region 23-FGAILS-28, which has been recognized early on to be of critical importance for IAPP amyloidogenicity [6, 8]. Our results from chemical shift analysis clearly indicate that the FGAILS region is part of a β-strand in our fibrils from hIAPP_COOH_. This finding is in line with the crystal structure based model, which proposes a β-strand starting from G24, [10] and with the serpentine model [30]. In contrast, other models suggest that this region may as well be part of a random coil loop [26–28]. Remarkable is the observation of a transient β-sheet in the aa region F23 to L27, as described for early oligomeric assemblies in an FTIR study by Buchanan et al. [29]. For mature fibrils, the authors report a change from β-strand to a disordered structure.

In the hIAPP_COOH_ fibrils, the conformation at position S29 is less constrained than at other sites, and a continuous β-strand may be adopted spanning the region from F23 to V32. A conformation in which aa residues F23 to V32 form a continuous β-strand is in reasonable agreement with the crystal structure based model [10] which predicts a β-strand for the GAILS segment.

Taken together, the chemical shift analyses of our NMR data as well as our NHHC transfers strongly support the hypothesis that the central segment FGAILS is able to adopt β-sheet conformation in mature IAPP_COOH_ fibrils. The comparison with literature data, indicates that this region, which is highly amyloidogenic, and critical for IAPP fibrillation, is the site of significant conformational variability in IAPP amyloid fibrils.

### Short kinks with higher flexibility connect β-strands in C-terminus

Residues S29 and G33/S34 showed more flexibility than the adjacent C-terminal β-strands. Thus, they probably form kinks that exhibit higher dynamics than the β-strands.

At positions G33/S34, all models presented suggest a β-sheet. Our data propose that this β-structure may be disrupted by the glycine residue at position G33 and the subsequent S34. As discussed above, S34 showed less intense peaks in 3D spectra than its neighboring residues. We deduce that it is in a less ordered state, in agreement with the possible formation of a kink at this position.

Our data indicate that β-structure dominates in the C-terminal sequence region from F23 to Y37. However, the β-structure is interrupted by kinks with higher flexibility and increased dynamics around S29 and G33/S34. Recent structures [65–67] or secondary structural analyses [68] of other amyloid fibrils also identify shorter β-sheets consisting of a minimum of 2 residues with kinks at almost every glycine residue. Thus, our secondary structure analysis agrees well with recent findings on amyloid structures.

For the first and last residue in the sequence, TALOS-N does not provide a backbone dihedral angle prediction. Thus, from TALOS-N results alone we cannot state if Y37 is still part of the last β-strand or not. We did however observe that its secondary chemical shifts were not typical of a β-conformation, and in 3D experiments it did not show intense peaks. This behavior indicates that it is not part of a β-sheet but has a higher degree of flexibility, a result which may well be due to the missing amide group in IAPP_COOH_.

### Structure of the N-terminus hIAPP(1–8)

Considering their high peak intensities, their particular chemical shifts, and their enhanced T_1ρ_ relaxation at weak spin-lock fields (ω_rf_<10 kHz), the N-terminal aa residues N3 to T6 appear to have a more dynamic behavior than the β-strand regions, presumably due to a less dense packing. We assume free side chain rotations about the Cα-Cβ bonds and potential restricted puckering motions of the loop which lead to partial averaging of the backbone torsion angles. Partial conformational averaging may also be responsible for the remarkably narrow lines. We underline the observation that the N-terminal residues showed the same conformation in two independently prepared samples. A further argument in favor of partial chemical shift averaging due to restricted conformational sampling is the finding that the Cα-Cβ cross peak of A5 was broadened beyond detectability upon lyophilization [26], or upon freezing the sample to temperatures around 100 K (Fig 5).

## Conclusion

We presented our results of solid-state NMR on recombinant, fully ^13^C, ^15^N-labeled human IAPP_COOH_ after fibrillation. Analysis of chemical shifts led to the conclusion that there are up to four β-strands per monomer present in fibrillar IAPP_COOH_. They are located at aa positions 8 to 20, 23 to 28, 30 to 32, and 35 to 36. The second and third β-strand can also form one single β-strand, as indicated by the observation of β-conformation for a fraction of the S29 residues.

Notable is the appearance of a β-strand in the structurally important FGAILS region IAPP(23-28), in our fibril preparations, which in this respect differ from some previous structural models of IAPP fibrils. Furthermore, we find the N-terminal aa residues IAPP(1-8) being in a well ordered conformation that exhibits a higher degree of flexibility than the β-strands. A comparison with an earlier solid-state NMR study on synthetic IAPP_CONH2_ indicates structural differences in the aa region L27 to S34, demonstrating the presence of polymorphism in IAPP amyloid fibrils.

## Supporting Information

S1 Fig PDSD spectra with short and long mixing times. A) Proton-Driven-Spin-Diffusion (PDSD) spectra recorded at a field of 18.8 Tesla with longitudinal mixing times of 20 ms (black) and 200 ms (red) and spinning speeds of 11 kHz and 12.5 kHz respectively. The VT gas temperature was set to 0°C in both experiments. Short mixing time (20 ms) provides mainly intra-residual peaks, while long mixing time (200 ms) at spinning close to rotational resonance condition shows many inter-residual peaks. Assignments shown are based on a number of experiments and brought together in this figure. Inter-residual cross-peaks connecting T4-A5 as well as S34 with its neighboring residues are indicated. B) Spectrum of the first FID of the 20 ms PDSD spectrum. C) Two cross-sections of the 200 ms mixing time spectrum as indicated by dashed lines in section A).
(TIF)

S2 Fig Reproducibility of fibril spectra. Overlay of two PDSD spectra acquired at a field strength of 14.1 T with an MAS spinning frequency of 11 kHz. Longitudinal mixing time was set to 50 ms in both experiments. The spectra were recorded on sample 1 (undiluted, uniformly ^13^C, ^15^N-labeled) and sample 2 (1 part uniformly ^13^C, ^15^N-labeled IAPP per 4 parts of unlabeled peptide). Blue peaks are from sample 1 (VT = 0°C) and pink peaks from sample 2 (VT = -10°C). Peaks are coincident for all residues but S29 and the sidechain Cō of I26. Differences are indicated by black circles. Cross-sections emphasize the intense peaks of A5 (A) and T4 (B) in both samples. N-terminal residues C2 - C7 show the same chemical shifts in both samples. These residues are marked by green circles.
(TIF)

S3 Fig 2D DREAM spectrum. Overlay of a 2D DREAM spectrum (green) and a PDSD spectrum with 20 ms longitudinal mixing (black). The DREAM spectrum was acquired at a field strength of 14.1 T with an MAS frequency of 22 kHz. The PDSD was acquired at a field strength of 18.8 T with an MAS frequency 11 kHz. In both experiments, the VT gas temperature was set to 0°C. Shown in red are two cross-sections of the DREAM spectrum. They show the impurities which were found in all spectra, but strongest appeared in the DREAM spectrum.
(TIF)

S4 Fig NHHC spectrum with 50 μs proton mixing compared to positions of C(i)-N(i+1) cross-peaks. The overlap with sequential ^13^C(i)-^15^N(i+1) peaks supports β-conformation in the peptide regions shown. Symbols indicate positions of sequential peaks using chemical shifts from current study. The black peaks are Cα(i) shifts correlated with N(i+1). In green are shown the Cβ(i) or Cγ(i) correlated with N(i+1). The filled symbols represent the FGAILS-region, IAPP(23–28), open symbols represent cross peaks predicted for residues belonging to residues of regions 8–20 and 29–36.
(TIF)

S5 Fig Comparison with chemical shifts from former ssNMR study. Overlay of PDSD spectrum (black) with peaks generated from chemical shifts as published in 2007 by Luca et al. [26] (blue squares). A 2 ppm correction to the shifts reported by Luca et al. was made before comparison as different referencing compounds were used in both studies. Our spectrum was acquired at a field of 18.8 Tesla at 11 kHz MAS and a VT gas temperature of 0°C. The longitudinal mixing time was set to 20 ms. In the experiments from Luca et al., the N-term, except of A5, was not labeled. No systemic deviation in between the chemical shift values of the two studies is found, rather a partial agreement and partial disagreement between the peaks. Major differences in chemical shifts are observed for residues spanning region L27-S34. These are displayed in colors and marked with the respective residue number. Circles belong to our peaks and the colored boxes indicate the corresponding peaks from the study of Luca et al.
(TIF)

S1 Table Experimental details. Sample 1 is fully ^13^C, ^15^N-labeled fibrillar IAPP. Sample 2 is fibrillar IAPP with 1 part ^13^C, ^15^N-labeled per 4 parts unlabeled peptide. Both samples were expressed and purified equally as described in methods part.
(PDF)

S2 Table Chemical shifts in ppm. Chemical shifts in ppm derived from solid-state NMR experiments and used for TALOS-N predictions and calculation of secondary chemical shifts. Small letters for cysteine residues indicate the oxidized disulfide bridge. The oxidation state is considered by TALOS-N routine. *The second Cβ chemical shift value of 63.7 ppm for S29 corresponds to an additional conformation as observed in the first sample (weak) and in the second sample (strong).
(PDF)

S3 Table TALOS-N backbone torsion angle predictions. Predicted backbone torsion angles for chemical shifts from S2 Table. The Classification describes the consensus of the torsion angle with database values. “Warn” means that there is no consensus in database matches. “Strong” indicates a major consensus in database matches.
(PDF)

## Abbreviations

aa: amino acid
AFM: Atomic force microscopy
DNP: Dynamic nuclear polarization
DREAM: Dipolar recoupling enhanced by amplitude modulation
E.coli: Escherichia coli
IAPP: Islet Amyloid Polypeptide
INEPT: Insensitive nuclei enhanced by polarization transfer
MAS NMR: Magic Angle Spinning Nuclear Magnetic Resonance
PDSD: Proton-driven spin-diffusion
RP-HPLC: Reversed-phase high-performance liquid chromatography
