# Supplementary material for: Structural Characterization of Fibrils from Recombinant Human Islet Amyloid Polypeptide by Solid-State NMR: The Central FGAILS Segment Is Part of the β-Sheet Core
Source: PLoS One. 2016 Sep 8;11(9):e0161243. doi: 10.1371/journal.pone.0161243 (PMC5015977; doi:10.1371/journal.pone.0161243)
Supplement: S1 Table — Sample 1 is fully 13C, 15N-labeled fibrillar IAPP. Sample 2 is fibrillar IAPP with 1 part 13C, 15N-labeled per 4 parts unlabeled peptide. Both samples were expressed and purified equally as described in methods part. (PDF) [file pone.0161243.s006.pdf]

Table S1, Experimental details<br>**Sample 1**										**Sample 2**	**DNP**
	INEPT 1D	PDSD 2D	SPC5_3 2D	DREAM 2D	NHHC 2D	NCaCB 2D	NCaCX 3D	NCaCB 3D	NCOCX DARR 3D	PDSD 2D	SPC5_2 2D
Mixing time		1) 20 ms<br>2) 200 ms			50 µs		80 ms		80 ms	50 ms	
^{1}H frequency (MHz)	600	800	600	600	600	600	600	600	600	600	600
MAS (Hz)	11000	1) 11000<br>2) 12500	11000	22000	11000	22000	11000	22000	14000	11000	8000
VT gas temperature	0°C	0°C	0°C	0°C	0°C	0°C	0°C	0°C	-10°C	-10°C	100 Kelvin
Transfer 1	HC-CP	HC-CP	HC-CP	HC-CP	HN-CP	HN-CP	HN-CP	HN-CP	HN-CP	HC-CP	HC-CP
Carrier (ppm)	55	55	40	57	110	120	120	120	120	90	135
Duration of 1st transfer	200 µs	200 µs	600 µs	200 µs	200 µs	400 µs	500 µs	400 µs	400 µs	200 µs	150 µs
Transfer 2		-	-	-	NH-CP	N-CA SPECIFIC CP	N-CA SPECIFIC CP	N-CA SPECIFIC CP	N-CO SPECIFIC CP		
Carrier (ppm)		-	-	-	110	55	55	50	170		
Duration of 2nd transfer					200 µs	1200 µs	1300 µs	1350 µs	1600 µs		
t1 increments	758	750	463	938	463	476	1172	417	537	758	512
t1 spectral width (kHz)	37.8	62.5	46	78.1	46	60	77.7	60	44	37.8	44
t2 increments		240	120	128	16	27	8	12	14	160	160
t2 spectral width (kHz)		40	22	18	4	3.6	2.4	2.4	2.4	33	38
t3 increments							18	18	15		
t3 spectral width (kHz)							4.5	4.5	3		
Number of scans	128	212	256	176	4928	2944	640	352	432	1008	32
Duration	< 1 h	63 h	24 h	24 h	84 h	144 h	216 h	254 h	216 h	187 h	7 h

S1 Table Experimental details.
Sample 1 is fully ^{13}C, ^{15}N-labeled fibrillar IAPP. Sample 2 is fibrillar IAPP with 1 part ^{13}C, ^{15}N-labeled per 4 parts unlabeled peptide. Both samples were expressed and purified equally as described in methods part.
