# Supplementary material for: Structural Characterization of Fibrils from Recombinant Human Islet Amyloid Polypeptide by Solid-State NMR: The Central FGAILS Segment Is Part of the β-Sheet Core
Source: PLoS One. 2016 Sep 8;11(9):e0161243. doi: 10.1371/journal.pone.0161243 (PMC5015977; doi:10.1371/journal.pone.0161243)
Supplement: S2 Table — Chemical shifts in ppm derived from solid-state NMR experiments and used for TALOS-N predictions and calculation of secondary chemical shifts. Small letters for cysteine residues indicate the oxidized disulfide bridge. The oxidation state is considered by TALOS-N routine. *The second Cβ chemical shift value of 63.7 ppm for S29 corresponds to an additional conformation as observed in the first sample (weak) and in the second sample (strong). (PDF) [file pone.0161243.s007.pdf]

S2 Table, Chemical shifts in ppm									
	residue	N	CO	Ca	Cb	Cg	Cd	Ce	Cz
1	K	123.0	174.1	54.6	32.8	25.1	29.7	41.9	
2	c	125.2	173.6	59.2	43.5				
3	N	114.2	176.6	51.3	39.1	176.6			
4	T	119.7	175.8	62.1	66.1	23.9			
5	A	119.3	176.4	51.0	26.8				
6	T	117.1	174.0	61.7	70.2	21.4			
7	c	120.3	173.1	53.2	48.7				
8	A	122.2	175.5	50.7	22.7				
9	T	119.1	172.5	61.8	70.3	21.9			
10	Q	125.8	174.3	54.4	32.2	34.2	179.5		
11	R	125.2	173.5	54.9	30.5	27.3	43.8		159.6
12	L	127.9	172.7	54.8	44.2	29.3	25.1		
13	A	129.4	174.5	51.4	21.2				
14	N	120.5	173.2	52.5	42.8	177.3			
15	F	117.1	173.2	56.6	42.7	137.9	131.6		
16	L	119.2	173.9	56.1	42.0	30.4	25.1/27.6		
17	V	120.7	174.5	60.7	35.8	21.5			
18	H	122.0	175.0	54.3	28.2	131.6			
19	S	124.0	174.0	55.9	64.5				
20	S	124.2	174.7	55.9	64.5				
21	N	127.3	173.0	54.2	36.4				
22	N	118.1	172.2	52.5	42.3				
23	F	121.1	173.7	57.1	40.2	137.8	131.4		
24	G	109.9	171.3	43.9					
25	A	127.9	176.8	50.4	21.8				
26	I	120.4	177.0	60.1	40.5	16.9/27.6	12.4		
27	L	121.5	173.6	54.2	44.5	29.3	23.7/26.8		
28	S	116.3	173.6	55.8	64.5				
29	S	117.5	173.7	58.9	61.7/63.7*				
30	T	114.4	171.4	61.3	74.0	21.3			
31	N	122.3	173.9	52.3	41.3	176.1			
32	V	121.7	174.8	60.5	34.6	20/22.2			
33	G	116.7	174.0	48.0					
34	S	113.1	171.7	58.4	68.4				
35	N	120.0	174.3	52.5	41.9	176.9			
36	T	114.4	173.3	60.7	71.4	21.5			
37	Y	128.5	180.7	60.0	39.5	129.1	132.8	117.8	156.1

S2 Table Chemical shifts in ppm.

Chemical shifts in ppm derived from solid-state NMR experiments and used for TALOS-N predictions and calculation of secondary chemical shifts. Small letters for cysteine residues indicate the oxidized disulfide bridge. The oxidation state is considered by TALOS-N routine. *The second Cβ chemical shift value of 63.7 ppm for S29 corresponds to an additional conformation as observed in the first sample (weak) and in the second sample (strong).
