# Supplementary material for: Structural Characterization of Fibrils from Recombinant Human Islet Amyloid Polypeptide by Solid-State NMR: The Central FGAILS Segment Is Part of the β-Sheet Core
Source: PLoS One. 2016 Sep 8;11(9):e0161243. doi: 10.1371/journal.pone.0161243 (PMC5015977; doi:10.1371/journal.pone.0161243)
Supplement: S3 Table — Predicted backbone torsion angles for chemical shifts from S2 Table. The Classification describes the consensus of the torsion angle with database values. “Warn” means that there is no consensus in database matches. “Strong” indicates a major consensus in database matches. (PDF) [file pone.0161243.s008.pdf]

S3 Table, TALOS-N backbone torsion angle predictions						
	residue	φ	ψ	σ_φ	σ_ψ	classification
1	K	-	-	-	-	None
2	C	-69,0	-31,7	9,8	8,8	Warn
3	N	-110,0	129,3	19,8	47,4	Warn
4	T	-66,3	138,5	9,2	13,2	Warn
5	A	-144,5	156,0	11,2	8,2	Strong
6	T	-100,8	134,6	15,8	11,3	Strong
7	C	-138,3	159,4	9,5	8,0	Strong
8	A	-135,6	140,7	12,2	10,0	Strong
9	T	-110,8	128,9	14,3	7,0	Strong
10	Q	-120,1	133,4	10,6	9,4	Strong
11	R	-97,0	124,5	9,9	10,9	Strong
12	L	-118,0	129,1	10,5	7,4	Strong
13	A	-117,9	132,1	10,0	6,6	Strong
14	N	-123,1	141,0	15,0	11,2	Strong
15	F	-136,2	143,5	12,3	11,1	Strong
16	L	-76,4	132,1	11,0	7,9	Strong
17	V	-126,5	134,3	11,6	11,5	Strong
18	H	-91,9	119,3	10,5	7,5	Strong
19	S	-105,5	118,0	10,8	24,8	Strong
20	S	-127,0	132,9	10,0	13,3	Strong
21	N	49,2	47,4	5,2	6,3	Warn
22	N	-131,4	156,0	15,1	12,5	Strong
23	F	-104,8	129,3	15,0	7,7	Strong
24	G	-113,7	148,8	19,0	10,6	Strong
25	A	-119,5	128,3	11,0	6,4	Strong
26	I	-111,7	134,2	12,4	10,7	Strong
27	L	-122,3	131,1	12,2	12,2	Strong
28	S	-125,8	133,6	10,0	9,6	Strong
29	S	50,1	52,3	4,5	8,5	Warn
30	T	-139,3	151,8	8,8	11,4	Strong
31	N	-112,0	129,5	14,6	12,5	Strong
32	V	-122,4	128,5	10,1	13,0	Strong
33	G	-133,3	168,7	45,2	34,9	Warn
34	S	-147,6	152,7	9,4	8,0	Strong
35	N	-128,2	142,2	18,2	10,9	Strong
36	T	-114,1	140,2	17,7	14,7	Strong
37	Y	-	-	-	-	None

S3 Table TALOS-N backbone torsion angle predictions.

Predicted backbone torsion angles for chemical shifts from S2 Table. The Classification describes the consensus of the torsion angle with database values. “Warn” means that there is no consensus in database matches. “Strong” indicates a major consensus in database matches.
